# Effectiveness of Anthropometric Measurements for Identifying Diabetes and Prediabetes among Civil Servants in a Regional City of Northern Ethiopia: A Cross-Sectional Study

**DOI:** 10.1155/2020/8425912

**Published:** 2020-04-07

**Authors:** Ataklti Gebertsadik Woldegebriel, Kiros Ajemu Fenta, Asfawosen Berhe Aregay, Abraham Desta Aregay, Nega Bezabih Mamo, Tewolde Woldearegay Wubayehu, Alemayehu Bayray, Afework Mulugeta

**Affiliations:** ^**1**^ Tigray Health Research Institute, Mekelle, Ethiopia; ^**2**^ School of Public Health, Mekelle University, Mekelle, Ethiopia

## Abstract

**Methods:**

The study involved a cross-sectional survey carried out from October 2015 to February 2016 among 1504 subjects aged from 18 to 75 years of age. Receiver operating characteristic (ROC) was used to select the most effective anthropometric cut-off point among waist circumference, waist-to-hip ratio, waist-to-height ratio, and BMI for identifying prediabetic and diabetes. Statistical significance was declared at *p* value of ≤0.05.

**Results:**

Waist circumference was found better for identifying diabetes (AUC = 0.69) and prediabetes (AUC = 0.63) in women, respectively. Waist-to-hip ratio was better identifying diabetes (AUC = 0.67) while waist circumference-to-height ratio was better identifying prediabetes (AUC = 0.63) in men compared to body mass index. The optimal cut-off point with maximum sensitivity and specificity of waist circumference for identifying diabetes and prediabetes was 83.5 cm and 82.9 cm in women, respectively. The optimal ut-off point with maximum sensitivity and specificity of waist-to-hip ratio for identifying diabetes and prediabetes was 0.97 and 0.82 in men, respectively.

**Conclusion:**

Waist circumference and waist-to-hip ratio exhibited better discriminate performance than BMI for identifying prediabetes and diabetes in women and men, respectively.

## 1. Background

In recent years, there was industrialization and great modification in the lifestyle and consequently life treating metabolic disorders encounter due to obesity and overweight which causes noncommunicable diseases such as diabetes, cardiovascular disease, and hypertension. Diabetes caused by hyperglycemia results from abnormal in insulin metabolism [1, 2]. Globally, there is a rapid growing of incidence of prediabetic and diabetes. The highest increase (156%) in the number of people with diabetes worldwide between 2017 and 2045 will occur in sub-Saharan Africa.

Diabetes mellitus is one of the four major noncommunicable diseases (NCDs) in Ethiopia. Ethiopia has the highest numbers of people with diabetes in Africa. According to International Diabetes Federation Atlas (IDFA) in 2017, the prevalence of diabetes ranges from 2.02% to 7.3%. The prevalence of prediabetes in Ethiopia was 12.6% in males and 28.2% in females with a total prevalence of 20.3% [3–5].

Obesity is an emerging problem and public health significance in sub-Saharan Africa including Ethiopia [6]. The distribution of fat in the body is measured by using simple, most practical, at a low cost, and widely used markers of obesity index like anthropometric measurements, such as body mass index (BMI), waist circumference (WC), waist-to-hip ratio (WHR), and waist-to-height ratio (WHtR) [7, 8]. Different previous studies showed varied prediction abilities of anthropometric measurements to predict prediabetes and diabetes [9–21]. A study conducted in Ethiopia indicated that WC was a predictor of CVD risk [22].

Study showed that the relation between BMI, WC, WHtR, and WHR differs across populations [23]. However, currently there are no anthropometric cut-off points for prediction of prediabetes in Sub-Saharan Africa. Although there is in genetics, ethnic geographic distribution, and biological difference, the cut-off points were simply adopted from western populations [24]. For the same level of age, gender, and body fat composition, the Ethiopian body mass index was 4.6 kg/m^2^ lower compared to Caucasians [25].

Hence, population specific anthropometric measurement is necessary to predict prediabetes and diabetics.

However, controversy remains regarding anthropometric indices to predict prediabetes and diabetes. Therefore, the present study shall help as a reference for providing accurate cut-off values of anthropometric measurements that could be used in future studies to predict prediabetes and diabetes in the Ethiopian population. Therefore, this study was intended to explore anthropometric indices in predicting the risk of prediabetes and diabetes.

## 2. Methods

### 2.1. Study Setting and Period

This study is a part from a study that has been published. The study was conducted in Mekelle, the largest city in northern Ethiopia with 7 subcities (“KifleKetema”), which is located around 780 km of north of the capital city of Ethiopia, Addis Ababa. The estimated population of Mekelle city was about 320,000 residents in 2016 [26], and the public servant in the city accounts about 5% of the total population [27]. The study period was from October 2015 to February 2016.

### 2.2. Study Design and Population

A cross-sectional study was conducted using the NCD profile database among public employees in Mekelle city. The baseline characteristics of the study population have been described in the previous publication [28]. Public employees in the city were estimated to be 16,000 [26, 27]. A multistage sampling followed by simple random sampling technique was employed to reach the study participants: adult male and female study population, aged between 25 and 64 years, excluding pregnant and breastfeeding women.

### 2.3. Measurements

Data collection and measurement were conducted in accordance with the standardized WHO NCD STEPS instrument version 3.2. The study followed a stepwise approach to collect sociodemographic data, anthropometric measurements, clinical measurements, and laboratory analyses of lipid profile and fasting blood glucose level using a standardized protocol [29–34].

### 2.4. Anthropometry

Prior to the start of the study, a stadiometer (Seca Germany) was calibrated using calibration rods. The subjects stood on the stadiometer with their four points (heel, calf, buttocks, and shoulder) held in a natural nonstretched position. The subjects, in bare feet, stood erect with their heels touching each other, and the height of the study subjects were measured to the nearest 0.1 cm.

A portable digital scale was validated using an object of a known weight every morning and between the measurements. Weight was measured to the nearest 0.1 kg after the subjects were removed their shoes and heavy cloths prior to weighing. BMI was calculated as weight in kilograms divided by height in square meters.

The subjects were then classified into four groups according to the WHO BMI cut-offs: “Underweight”: BMI < 18.5  kg/m^2^, “Normal weight”: BMI = 18.5–24.9 kg/m^2^, “Overweight”: BMI = 25–29.9 kg/m^2^, and “Obese”: BMI ≥ 30 kg/m^2^.

Using a flexible nonelastic measuring tape, the waist circumference was measured by passing the measuring tape through the midway between the lowest costal margin at the mid-clavicle line and the anterior superior iliac spine, at the end of normal expiration.

The measurement of hip circumference was conducted stand up straight and wrap a tape measure around the level of the widest part of the hip, at the level of the greater trochanter with the subjects wearing a pant. In order to ensure the measurement accuracy, all anthropometric measurements were performed in triplicate and then the average value was used for further analyses. WHR was calculated as waist circumference divided by hip circumference, both measured to the nearest 0.1  cm using a steel retractable tape. WHtR was calculated by dividing the first measurement (waist circumference) by the second measurement (hip circumference).

Blood pressure (BP) was measured using a digital measuring sphygmomanometer (HEM-7200, OMRON, and Kyoto, Japan), three times on the left right upper arm with participants sitting after resting for at least 5 min with at least three-minute interval between measurements. Systolic blood pressure (SBP), diastolic blood pressure (DBP), and pulse per minute were recorded, and the arithmetic mean of the second and third readings of systolic and diastolic BP was considered for analyses.

### 2.5. Biochemical

For biochemical measurements, first of all, the study participants were given instruction to wait in the fasting condition for at least eight hours. Forty microliters of capillary blood was collected from the finger tip for biochemical measurement (glucose, total and HDL cholesterol, triglycerides, and HbA1c) and analyzed using standardized portable analyzers (Accu-Check Performa, Roche Diagnostics, Indianapolis, IN, USA, for glucose; cobas b 101, Roche Diagnostics, Indianapolis, IN, USA, for total and HDL cholesterol, triglycerides, and HbA1c; Hb 201+, HemoCu, Ängelholm, Sweden, for haemoglobin).

### 2.6. Data Quality Management

To ensure data quality and consistency, the questioner in Tigrigna was translated back to English to maintain quality, and to estimate the time required for collecting data, a pretest was conducted on 5% of the total sample size to check the feasibility of the data collection process. Questioners were revised based on the pretest, and the time required to fill one questioner was determined.

### 2.7. Data Processing and Analysis

The study aims to examine the effectiveness of anthropometric measurements on identifying prediabetes and diabetes and their cut-off point. Completeness and consistency of the data were checked before commencing the analysis. Analysis was carried out using STATA software package version 11. Descriptive statistics using frequencies and proportions were used to summarize variables. Data with normally distributed parameters were presented using mean ± SD. ROC curve of each anthropometric measurement was calculated at 95% confidence intervals in a 2-sided test compared to the golden standard fasting blood sugar (FBS).

Receiver operating characteristic (ROC) analysis was used to compare discrimination ability and determine optimal cut-off values. Sensitivity and specificity were calculated based on cut-off values, which were estimated using the maximized Youden index.

### 2.8. Anthropometric Measurement Cut-Off Values for the Statistical Analysis

Body mass index (BMI): weight in kilograms divided by height in meters squared [35] underweight: BMI < 18.5 kg/m^2^; overweight: BMI 25–29.9 kg/m^2^; obesity: BMI ≥ 30  kg/m^2^. Increased waist circumference: >94 cm for men; >80 cm for women [25]. Waist-hip ratio: ≥0.90 for men; ≥0.85 for women [36].

### 2.9. Prediabetes, Diabetes, Hypertension, and Lipid Profile Cut-Off Values

Prediabetes: FBG 100–126 mg/dL. Diabetes: FBG ≥ 126 mg/dL [26, 37]. Hypertension: SBP  ≥ 140 mmHg or DBP ≥ 90 mmHg or on antihypertensive medication [27], HDL cholesterol  < 40  mg/dL for men; <50 mg/dL for women, triglycerides ≥ 150 mg/dL, and raised LDL cholesterol ≥ 130 mg/dL [28].

## 3. Results

### 3.1. Basic Characteristics of the Study Subjects

Out of the 1504 study participants in the 18 public offices, 857 (56.98%) of them were male employees. The mean age of the study subjects were 39.3 years with a range of 18 to 75 years. The majority of the respondents (71.75%) were in the age group of 24–44 years. More than half (55.5%) of the study participants have less than 1000 individual gross annual income, SD per adult (Table 1).

The mean (SD) body mass index, waist circumference, waist circumference-to-hip ratio, and waist circumference-to-height ratio was 23.03 (0.65) kg/m^2^, 82.9 (0.09) cm, 0.84 (0.09), and 0.51 (0.01), respectively, in females, whereas the mean (SD) body mass index, waist circumference, waist circumference-to-hip ratio, and waist circumference-to-height ratio was 22.1 (6.02) kg/m^2^, 0.88.3 (0.36) cm, 1 (0.64), and 0.52 (0.09), respectively, in males (Table 2).

### 3.2. Anthropometric Measurements and Receiver Operating Characteristic Curve to Predict Diabetes

**Figure 1:**
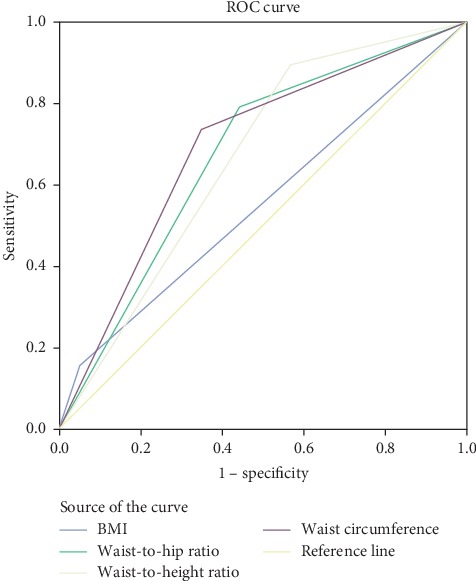
Receiver operating characteristic curve of body mass index (BMI), waist circumference (WC), waist-hip ratio (WHR), waist-to-height ratio (WHtR), and waist-to-square height ratio (WHt2R) to diabetes mellitus in female.

The AUC of the ROC analyses showed that the area under the curve to predict diabetes using waist circumference was higher in women 0.69 (95% CI: 0.577–0.812), followed by 0.67 (95% CI: 0.561–0.786) for waist-hip ratio, 0.66 (95% CI: 0.564–0.769) for waist-to-height ratio, and 0.52 (95% CI: 0.394–0.654) for BMI, respectively, whereas the AUC of the ROC analyses in men showed that there were no single anthropometric index that had consistently higher AUC value than the others. However, the area under the curve to predict diabetes using waist-to-hip ratio had relatively higher area under the curve in men than other anthropometric measurements with 0.61 (95% CI: 0.537–0.675) (Table 3 and Figure 1).

### 3.3. Anthropometric Measurements and ROC Curve to Predict Prediabetes

The AUC of the ROC analyses showed that the area under the curve to predict prediabetes using waist circumference-to-height ratio was 0.631 (95% CI: 0.588–0.675), which had relatively higher area under the curve in predicting prediabetes in men. However, the AUC of the ROC analyses to predict prediabetes using waist circumference had relatively higher area under the curve in women (AUC = 0.623; 95% CI: 0.537–0.78). BMI was with lower accuracy in predicting diabetes and prediabetes for both genders (Table 3 and Figure 1).

### 3.4. Optimal Cut-Off Points of Anthropometric Parameters to Identify Prediabetes

**Figure 2:**
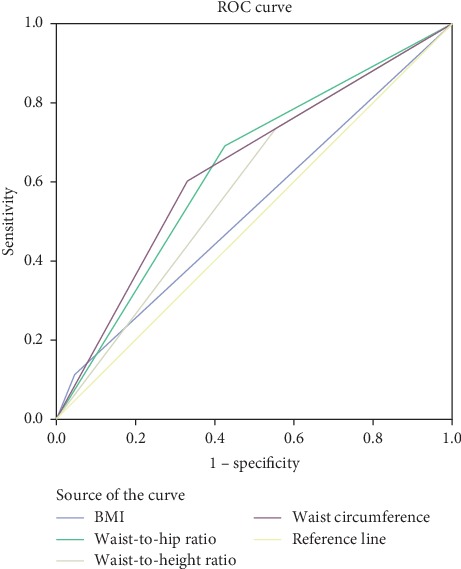
Receiver operating characteristic curve of body mass index (BMI), waist circumference (WC), waist-hip ratio (WHR), waist-to-height ratio (WHtR), and waist-to-square height ratio (WHt2R) to prediabetes in female.

**Figure 3:**
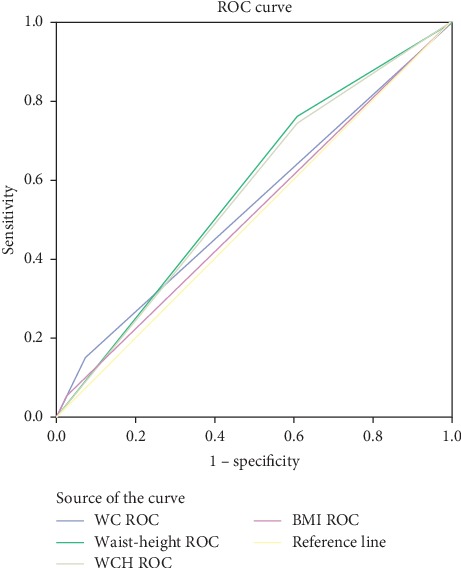
Receiver operating characteristic curve of body mass index (BMI), waist circumference (WC), waist-hip ratio (WHR), waist-to-height ratio (WHtR), and waist-to-square height ratio (WHt2R) to diabetes mellitus in male.

**Figure 4:**
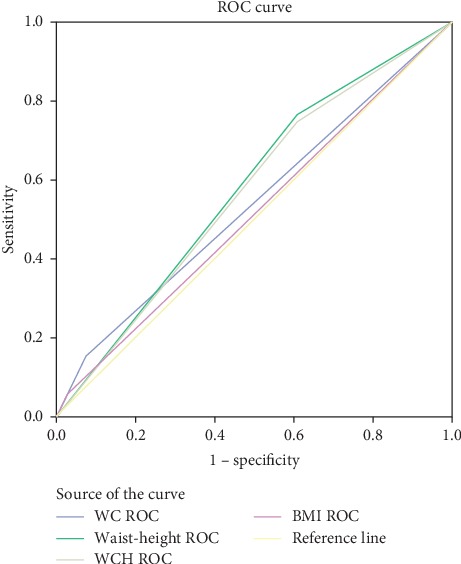
Receiver operating characteristic curve of body mass index (BMI), waist circumference (WC), waist-hip ratio (WHR), waist-to-height ratio (WHtR), and waist-to-square height ratio (WHt2R) to prediabetes in male.

The optimal cut-off values for predicting those prediabetes using sensitivity and specificity for the obesity index in ROC analysis are shown in Table 4. The estimated optimal cut-off points for BMI to predict prediabetes were ≥22.21 kg/m^2^ in men and ≥21.92 kg/m^2^ in female, whereas WC was 87.24 cm in men and 83.5 cm in women. Similarly, the estimated optimal cut-off points for WHR to predict prediabetes were 0.95 in men and 0.82 in female, and WHtR was 0.51 in men and 0.50 in women (Figures 2, 3, 4).

### 3.5. Optimal Cut-Off Points of Anthropometric Parameters to Identify Diabetes

The optimal cut-off value for predicting diabetes using sensitivity and specificity for obesity index in ROC analysis is shown in Table 4. The cut-off value for BMI for predicting diabetes was ≥23.02 kg/m^2^ in men and ≥ 20.5 kg/m^2^in women. The estimated optimal cut-off point for WC was 88.6 cm in men and 82.9 cm in women, WHR was 0.97 in men and 0.86 in women, and WHtR was 0.52 in men and 0.51 in women.

## 4. Discussion

Obesity is a major risk factor for developing diabetes. Especially, visceral or abdominal obesity increased the risk of metabolic conditions such as diabetes. This study suggests that waist circumference identifies better for prediabetes and diabetes in women. However, waist circumference-to-height ratio and waist-to-hip ratio identify better for prediabetes and diabetes in men. The optimal cut-off point for BMI in both sexes and WC in men for identifying prediabetes and diabetes was lower than optimal cut-off point stated by the World Health Organization.

Waist circumference (WC) for identifying prediabetes and diabetes was insignificant in men. The finding of this study is concurrent with the population-based cohort study that showed BMI and WC were not significant with incidence of diabetes in men [17]. Another study reported from Iran showed that association of WC with diabetes was higher in women than in men [38]. A study in Chinese adults showed that WC was a good marker for diabetes [35].

A study on cardiovascular risk in Ethiopia showed that fasting plasma glucose is most strongly associated with WC among women [22]. A study among white and black American adults showed that waist circumference was the best discriminators among white females [21]. This is an indication that WC can be used as a marker to identify prediabetes and diabetes.

This study evidenced that BMI had lower accuracy in predicting diabetes and prediabetes in both genders. A population-based cohort study showed that BMI was not a significant risk factor for prediabetes [17]. Another study showed that BMI was a good predictor of prediabetes for both sexes [9]. Among Ethiopian, Malaysian, and Thai, body fat percent was reported to be under estimated by BMI-based Caucasian predication equation [25]. The possible cause of the inferiority of BMI compared to WC might be that BMI cannot differentiate the visceral adiposity.

This study showed waist-to-hip ratio and waist-to-height ratio for identifying diabetes and prediabetes in men. A similar study from South India showed that waist-to-hip ratio was not a sensitive marker for females to predict diabetes [11]. Another study from Indonesia revealed that WtHR predicts prediabetes [39]. Another study from Chinese population showed that the risk of prediabetes increased significantly with increasing WC for both genders [14].

A study among white and black American adults showed that WHtR and WHR were the best discriminators among white females, whereas WHR was the best discriminator among black females [21]. In the literature, there is almost undisputed agreement on the association of central obesity measures with incident of diabetes in both sexes.

The variations of these study findings with the other studies might be attributable due to variations in population distinctiveness, cultural dynamics, ethnic groups, physical activities, sampling technique, methods of data collection, and differences in operational definitions.

This study raveled that the BMI cut-off point for identifying prediabetes was ≥ 23 kg/m^2^ in men and ≥20.5 kg/m^2^ in women and for identifying diabetes was ≥22.21 kg/m^2^ in men and ≥21.92 kg/m^2^ in women. The findings of the study are lower than the WHO recommendation as 25 kg/m^2^ in both genders.

This finding was close to the report from a study done in India, which showed the cut-off point of BMI for predicting prediabetes was 22.8 kg/m^2^ in boys and 20.5 kg/m^2^ in girls [9]. A study among Japanese showed BMI ≥ 23 is a risk factor for insulin resistance and diabetes [40]. Ethiopians have 4.6 kg/m^2^ lesser fat composition BMIs compared to the similar age of Caucasians [25]. The reason might be due to slender-like body posture which contains elevated body fat with lesser BMI.

The optimal cut-off points of WC for identifying diabetes were 88.6  cm and 83.5  cm for men and women, respectively. The optimal cut-off points of WC for identifying prediabetes were 87.2  cm and 82.9  cm for men and women, respectively. It is lower than the WHO standard, and its implication on the diabetes/prediabetes screening is that substantial portion of individuals with diabetes and prediabetes might have been undetected.

In the current study, cut-off points of different anthropometric measures using Youden's index give equal weight to both the sensitivity and specificity. Based on our data were identified the values of the WC that best balanced sensitivity and specificity. This decision rule accommodates the desire to prevent a significant risk of diabetes and prediabetes and the cut-off points identify risk factors with sensitivity greater than 83.3% and specificity greater than 40% in men and sensitivity greater than 86% and specificity greater than 46% in women, respectively.

In men, waist-to-hip ratio for identifying diabetes (AUC = 0.67) is with sensitivity greater than 66.3% and specificity greater than 33.3%. It can offer an alert about the practical boundary for initiating intervention to prevent and control the increase in the risk factor of prediabetes and diabetes as early as possible and redefining population-wise cut-off points.

The result showed that the WC and BMI cut-off point for women with diabetes is lower compared to prediabetes, which implies that higher WC gives lower risk of developing prediabetes similarly with the community epidemiological survey with the increase of the BMI cut-off point; the screening sensitivity for prediabetes is decreasing [41]. However, in a population-based study in women, the BMI cut-off point for predicting prediabetes was similar to the one for diabetes, but WC cut-off point was considerably higher [42]. The difference might be due to variation in physical activities, study setting, and socioeconomical characteristics.

## 5. Conclusion

We observed that WC was found to be better for identifying prediabetes and diabetes among women. WHR was found to be better for identifying prediabetes and diabetes among men. We observed that the identifying powers of BMI were lower in diagnosis of the incidence risk of prediabetes in men and diabetes in both sexes. The cut-off points of BMI for both sexes and WC in men were lower than cut-off points stated by the World Health Organization.

## Figures and Tables

**Table 1:** Sociodemographic characteristics of anthropometric indexes to detect diabetes mellitus to identify prediabetes and diabetes in public servants in Mekelle, Northern Ethiopia, 2016 (*N* = 1504).

Variables	Men (*n* = 857)	Women (*n* = 647)
*Age*		
25–34	183 (31.3%)	157 (40.4%)
35–44	215 (36.8%)	136 (35%)
45–54	144 (24.6%)	68 (17.5%)
55–64	43 (7.4%)	28 (7.2%)
*Educational status*		
Less than and primary school	69 (4.6%)	58 (3.8%)
Secondary and high school	92 (6.1%)	53 (3.5%)
College university degree	430 (28.5%)	433 (28.8%)
Postgraduate	257 (17%)	111 (7.3%)
*Marital status*		
Never married	25.3%	23.5%
Currently married	70.7%	59.6%
Separated/divorced/widowed	4.0%	16.9%
*Religion*		
Orthodox-Christians	95.5%	95.2%
Muslims	2.9%	2.4%
Other christians	1.6%	2.3%
*Annual income, USD per adult*		
<500	21.0%	31.9%
500–1000	28.3%	29.9%
1001–1500	21.7%	17.5%
>1500	29.0%	20.7%

**Table 2:** Physical and laboratory characteristics of anthropometric indexes to detect diabetes mellitus to identify prediabetes and diabetes in public servants in Mekelle, Northern Ethiopia, 2016 (*N* = 1504).

Anthropometric indices and cardiovascular risk factors	Men (*n* = 857)	Women (*n* = 647)
Height (cm)	1.690 ± .12	1.52 (0.29)
Weight (kg)	67.171 ± 3.59	56.85 (12.04)
BMI (kg = *m*2)	23.034 ± .65	22.1 (6.02)
WC (cm)	88.35 ± 0.36	82.86 (12.74)
HIP (cm)	99.283 ± 5.36	98.19 (9.73)
WHpR	1.000 ± 64	0.84 (0.09)
WHtR	0.51 ± 0.002	0.52 (0.09)
Systolic BP (mmHg)	124.221 ± 5.85%	114.55 (16.08)
Diastolic BP (mmHg)	81.81 ± 9.97%	77.49 (9.59)
Prediabetics	154 (19.3%)	48 (7.7%)
Diabetes	100 (5.6%)	21 (3.3%)
TCHO mg/dL	173.98 ± 11.710	174.20 (38.28)
TG mg/dL	175.12 ± 9.81	224 (12.07)
HDL mg/dL	26.09 ± 17.71	30.71 (2.21)
LDL mg/dL	77.435 ± .31	64.72 (5.31)
LDL (>150)	21 (2.1%)	24 (3.5%)
HDL (<40) for male or (<50) for female	665 (79.9%)	472 (80.3%)

**Table 3:** AUC for various anthropometric indices in men and women of anthropometric indexes to detect diabetes mellitus to identify prediabetes and diabetes in public servants in Mekelle, Northern Ethiopia, 2016 (*N* = 1504).

Female				
Variables	Waist circumference	Waist-hip ratio	Waist-height ratio	Body mass index
Diabetes mellitus	0.69 (0.577–0.812)	0.67 (0.561–0.786)	0.66 (0.564–0.769)	0.52 (0.394–0.654)
Prediabetes	0.62 (0.537–0.78)	0.57 (0.477–0.658)	0.58 (0.496–0.661)	0.59 (0.44–0.619)
Male		0.57 (0.494–0.638)	0.61 (0.537–0.675)	0.52 (0.394–0.654)
Diabetes mellitus	0.52 (0.394–0.654)	0.54 (0.49–0.589)	0.57 (0.49–0.6310)	0.50 (0.453–0.551)
Prediabetes	0.50 (0.453–0.551)	0.57 (0.494–0.638)	0.61 (0.537–0.675)	0.52 (0.394–0.654)

**Table 4:** The AUC and diagnostic performance of anthropometric indexes to detect diabetes mellitus to identify prediabetes and diabetes in public servants in Mekelle, Northern Ethiopia, 2016 (*N* = 1504).

Anthropometric indexes	Sex	AUC (95% Cl)	Sensitivity (%)	Specificity (%)	Cut-off points
*Diabetes*					
WC (cm)	F	0.69	83.3	40.0	83.5
	M	0.52	64	36	88.6
WCH	F	0.67	97.22	37.08	0.86
	M	0.57	66.7	33.3	0.97
WCHt	F	0.66	82	42	0.51
	M	0.61	78	37	0.52
BMI (kg/m2)	F	0.52	59	40	22.09
	M	0.52	56	42	23.02

*Prediabetes*					
WC	F	0.62	80	46	82.90
	M	0.50	48	30	87.24
WCH	F	0.57	69	41	0.82
	M	0.54	66	31	0.95
WCHt	F	0.58	57	47	0.5
	M	0.57	52	44	0.51
BMI (kg/m2)	F	0.59	69	41	21.92
	M	0.57	64	40	22.21

## Data Availability

The data used to support the findings of this study are available from the corresponding author upon reasonable request.

## Abbreviations

BMI: Body mass index
WC: Waist circumference
WHR: Waist-to-hip ratio
WHtR: Waist-to-height Ratio
kg: Kilogram
m^2^: Meter square
cm: Centimeter
NCD: Noncommunicable disease
T2DM: Type two diabetes mellitus
HbA1c: Haemoglobin 1c
WHO: World Health Organization
AOR: Adjusted odds ratio.
